# Supplementary figures and images for: Cofactor and glycosylation preferences for in vitro prion conversion are predominantly determined by strain conformation
Source: PLoS Pathog. 2020 Apr 15;16(4):e1008495. doi: 10.1371/journal.ppat.1008495 (PMC7185723; doi:10.1371/journal.ppat.1008495)

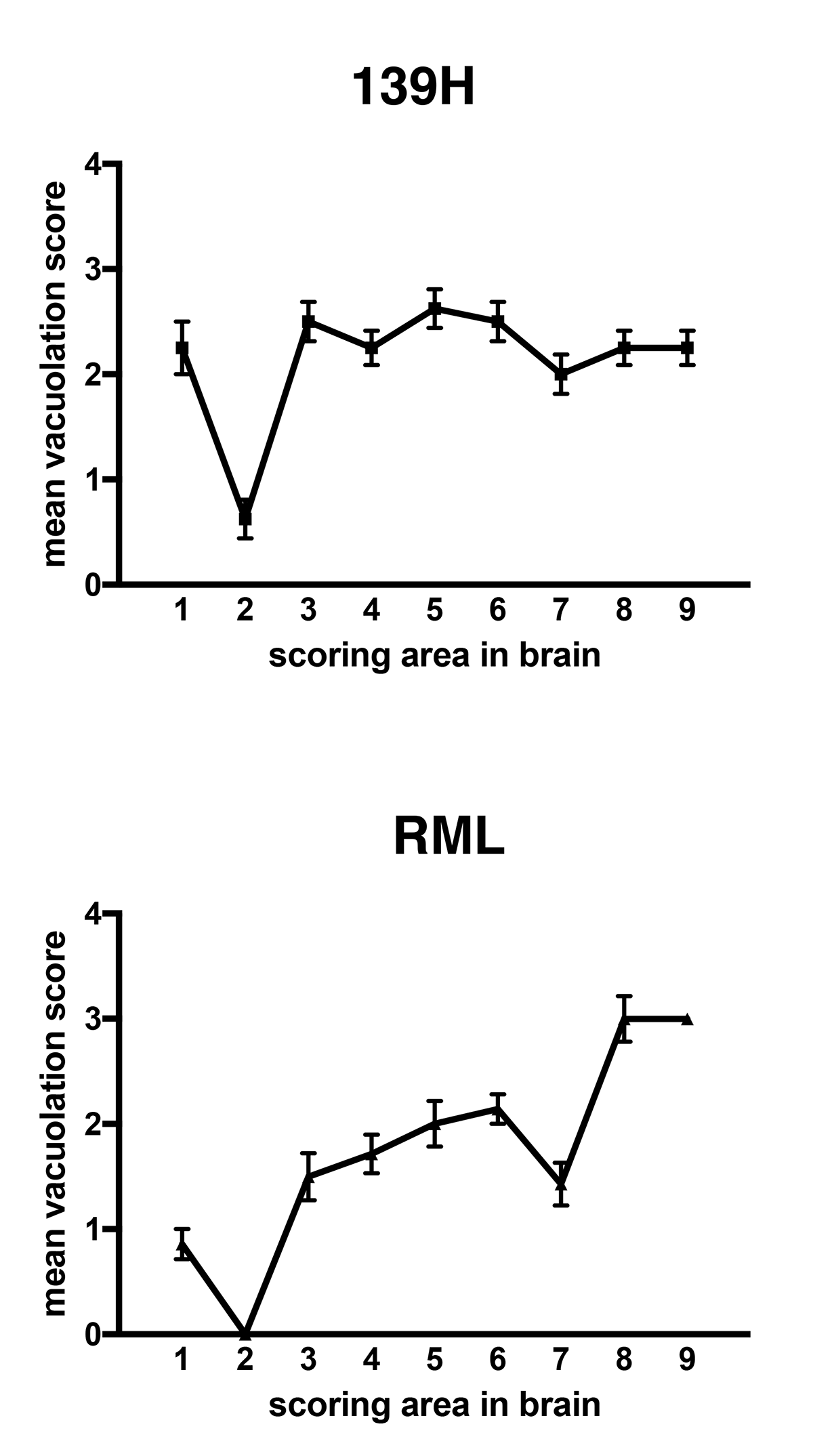

Supplement: S1 Fig — Lesion profiles of bank voles (M109 genotype) infected with 139H strain compared with RML strain, following third passage. The specific brain scoring areas analyzed were: (1) medulla, (2) cerebellum, (3) superior colliculus, (4) hypothalamus, (5) thalamus, (6) hippocampus, (7) septum, (8) retrosplenial and adjacent motor cortex, and (9) cingu- lated and adjacent motor cortex. (TIF) [file ppat.1008495.s001.tif]

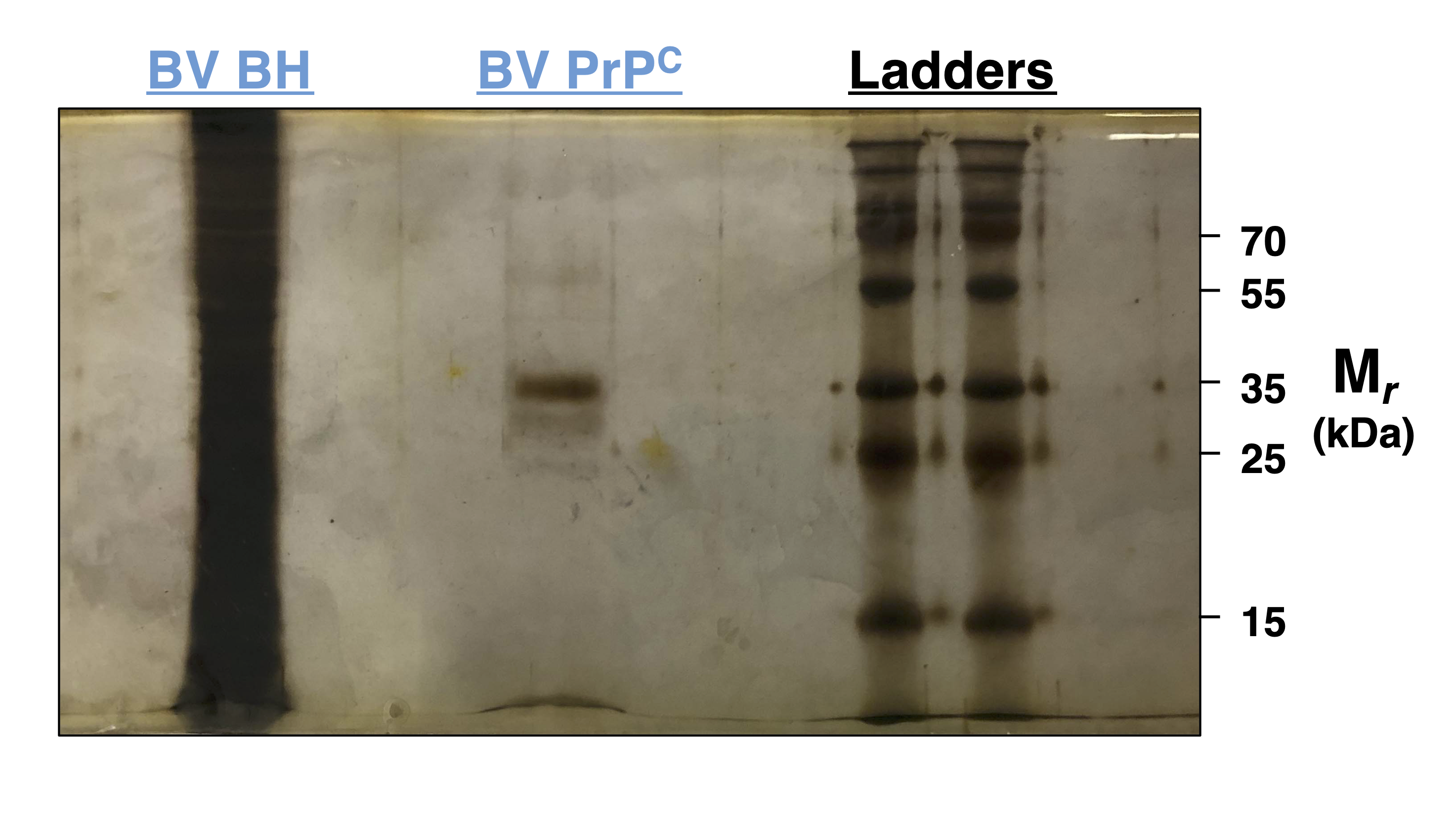

Supplement: S2 Fig — Twelve percent SDS/PAGE showing (from left to right): crude, detergent-solubilized bank vole brain homogenate (BV BH); immunopurified BV PrPC from bank vole brains; and molecular weight markers (ladders). (TIF) [file ppat.1008495.s002.tif]

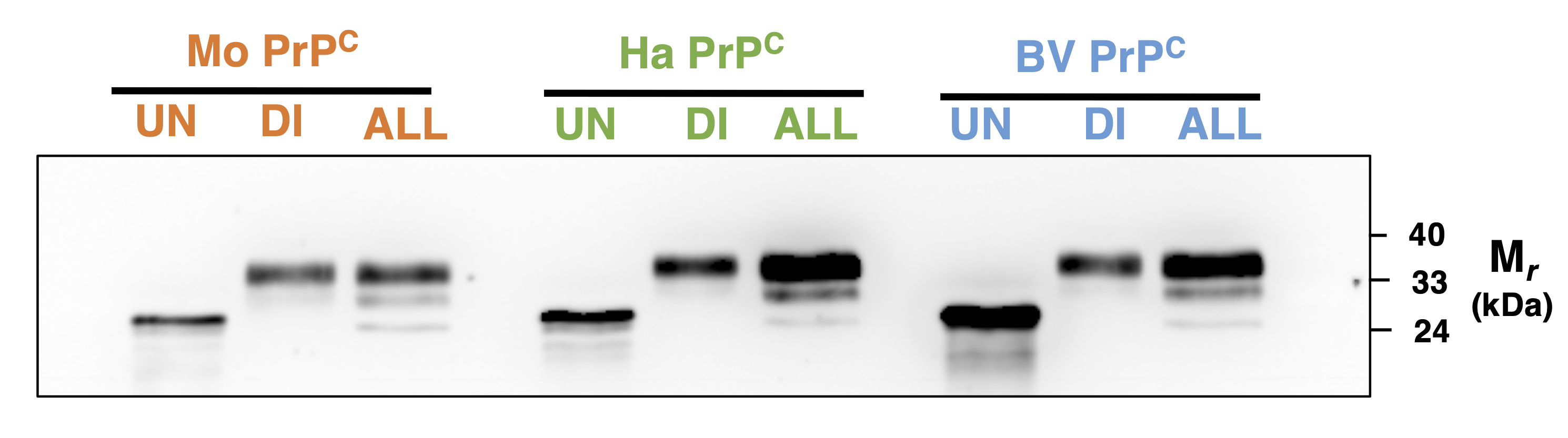

Supplement: S3 Fig — Western blot showing partially purified PrPC substrates from the indicated species that are used in sPMCA reactions. UN, PrPC substrate created by enzymatic deglycosylation of the DI substrate; DI, PrPC substrate eluted off the wheat-germ agglutinin column containing primarily diglycosylated PrPC; ALL, PrPC substrate containing all three glycoforms. (TIF) [file ppat.1008495.s003.tif]

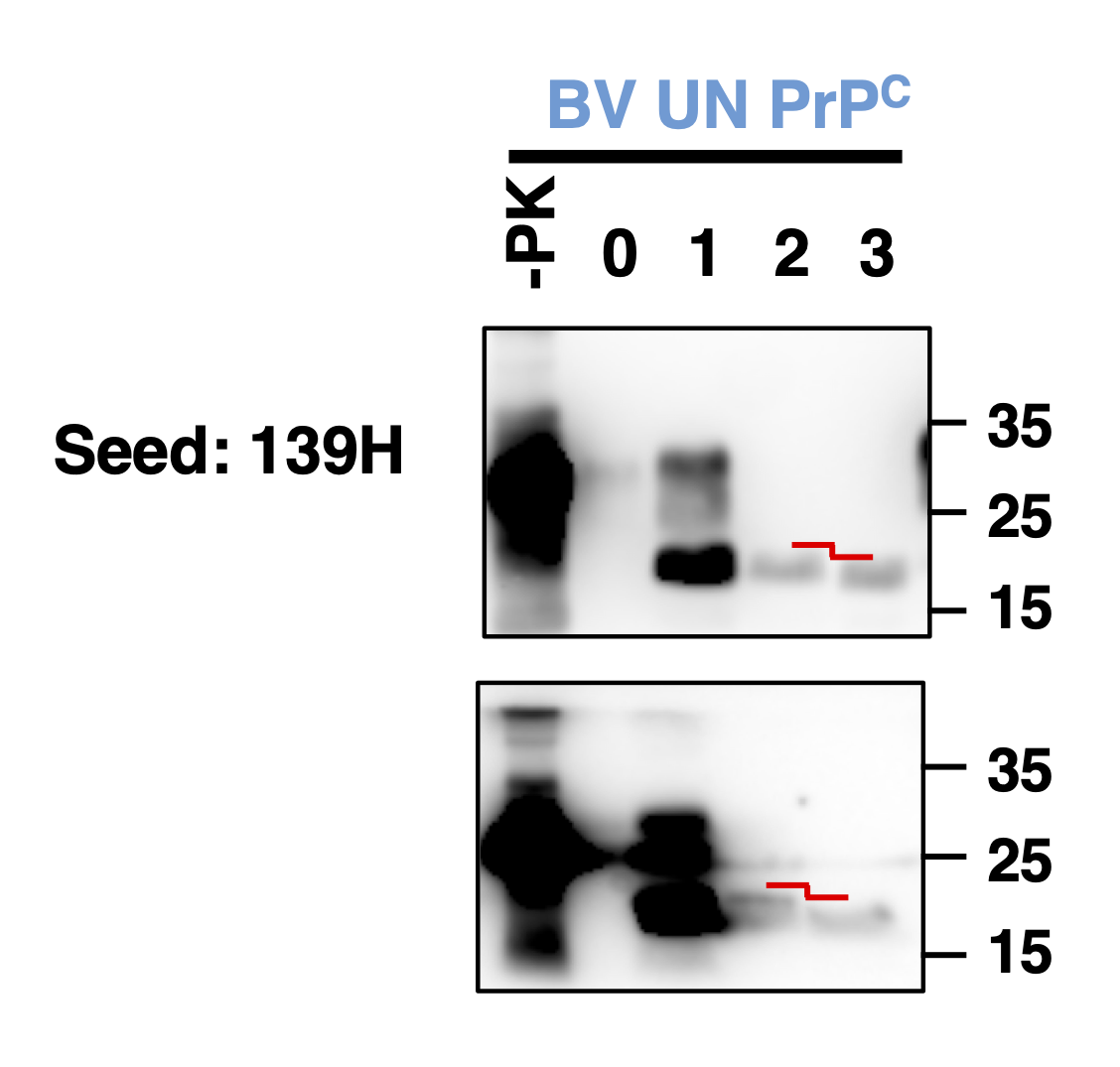

Supplement: S4 Fig — Western blots showing additional three-round sPMCA reactions demonstrating the MW shift observed in Fig 6, row 4, righthand column. The red lines highlight a shift in the apparent MW of the day three sample. Day 0 samples are a seeded reaction not subject to sonication. -PK = samples not subjected to proteinase K digestion; all other samples were proteolyzed. (TIF) [file ppat.1008495.s004.tif]

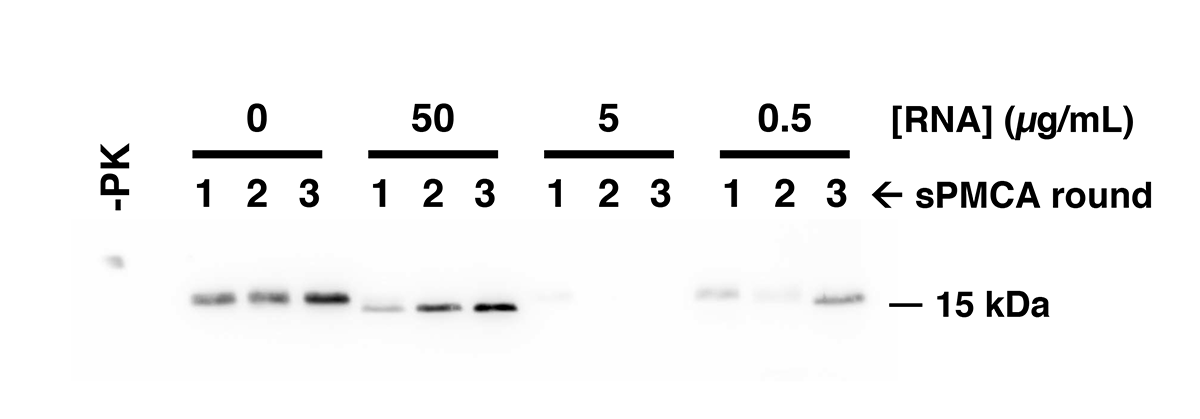

Supplement: S5 Fig — Three-round sPMCA reactions using mouse recombinant (rec)PrP substrate, mouse cofactor recPrPSc seed, and purified phospholipid cofactor were performed as previously described[16], in the presence of varying concentrations of synthetic poly(A) RNA, as indicated. In the absence of RNA, cofactor PrPSc maintains an ~18 kDa PK-resistant core during all 3 rounds of sPMCA. At [RNA] = 0.5 μg/mL, the PK-resistant core appears to shift stepwise to ~16 kDa between rounds 1–3; at [RNA] = 5 μg/mL, PrPSc propagation appears to be completely inhibited; and at [RNA] = 50 μg/mL, the PK-resistant core appears to shift to ~16 kDa immediately during the first round of sPMCA. Thus, addition of RNA appears to either (1) inhibit propagation and/or (2) force conformational adaptation of cofator PrPSc into a self-propagating conformer (similar to non-infectious protein-only PrPSc) in a concentration-dependent manner. (TIF) [file ppat.1008495.s005.tif]
