# Supplementary material for: Cofactor and glycosylation preferences for in vitro prion conversion are predominantly determined by strain conformation
Source: PLoS Pathog. 2020 Apr 15;16(4):e1008495. doi: 10.1371/journal.ppat.1008495 (PMC7185723; doi:10.1371/journal.ppat.1008495)
Supplement: S1 Table — Table showing RNA levels in RNA minipreps from untreated (-RNase) or RNase-treated (+RNase) crude 10% brain homogenate substrates from various species, as measured by spectroscopy. (DOCX) [file ppat.1008495.s006.docx]

| Crude BH Sample | RNA Concentration (ng/μL) |
| --- | --- |
| BV BH -RNase | 24.5 |
| BV BH + RNase | 2.9 |
| Mo BH - RNase | 27.9 |
| Mo BH + RNase | 1.2 |
| Ha BH - RNase | 41.0 |
| Ha BH + RNase | 1.5 |
