# Supplementary material for: Cofactor and glycosylation preferences for in vitro prion conversion are predominantly determined by strain conformation
Source: PLoS Pathog. 2020 Apr 15;16(4):e1008495. doi: 10.1371/journal.ppat.1008495 (PMC7185723; doi:10.1371/journal.ppat.1008495)
Supplement: Attachment — Submitted filename: Response to Reviews.docx [file ppat.1008495.s007.docx]

**Response to Reviews:**

We sincerely thank the editors and all three reviewers for their time and effort in reading our manuscript and for making constructive and helpful suggestions for improvement. We have modified the manuscript accordingly, and provided a point-by-point response below (*in italics*).

**Reviewer #1:**

1. The chief concern (this reviewer) has is the use and interpretation of dictation and that strain seeds are dictators. It could be viewed, as nicely acknowledged in the Discussion, that the (mouse or hamster-adapted) strains (of scrapie) have arisen from environments that have led to the strain seed conformation selection and fixation (usually involving serial passage). In that respect, have the substrate environments, usually brain tissue, not had part in the dictating that gave rise to the strains? So to this reviewer casting prion strain/seed, vs. substrate properties, as the dictator seems overly strained.

What is elegantly shown in that the interaction of seed, substrate conformations, glycosylation, and cofactors (all found cellular environments) determine the evolution of variants and eventual dominance of prion strains (the classic example used, 139A and H). [Perhaps the most robust naturally occurring example in BSE, which can ‘dictate’ across species boundaries.] So (to this reviewer) the process of strain origin and dominance seems analogous to fitness selection without genetic mutation to cement the process--certainly remarkable and why the protein only hypothesis is so significant (the point the ms. addresses). Therefore (again, to this reviewer), the work is excellent, but the emphasis on seed dictation is overstated.

*We agree, and have made appropriate changes to title, abstract, and main text to avoid overstating our interpretation. Specifically, we have replaced the word “dictate” with “select” or “plays a dominant role in determining.”*

2. The Results section contains extensive use of acronyms. While defined initially, the density of these can make sentences hard to interpret without back-searching to be sure of the what is being studied/tested. More information to help the reader with recalling the identity of the strains when mentioned would help the reader put the experiment premise and result in context without backtrack reading to remember what e.g. 139A, Me7, 22A and 301C are again. Just adding mouse helps a lot.

*We apologize for the density of our original text and have substituted full terms for most of the acronyms throughout the revised text.*

**Reviewer #2:**

1. The PMCA amplifications of the mouse prion strains in Fig 2 are not convincing in that the banding profiles are a rather faint and blurry and do not differ much at all from the unsonicated “0 round” samples whether the substrate BH for the reaction was RNase treated or not. The data in subsequent figures, using purified PrPC, are much more convincing. It would help the reader if the authors provided more perspective on why we should believe what is going on in Fig 2.

*We apologize that the quality of the PMCA data in Fig 2 as elsewhere. In our experience, PMCA experiments like this one using crude brain homogenates that involve pre-incubation @ 37^o^C for extended periods prior to addition of PrP^Sc^ seed (including the untreated mock control sample) often don’t amplify as well as experiments using fresh brain homogenates not subjected to pre-heating or reconstituted reactions (such as those shown in subsequent figures). This effect may be due to enzymes the crude brain homogenate that either degrade or misfold PrP^C^, and appears to affect mouse PMCA a bit more than hamster PMCA perhaps because the hamster strains that we used amplified a bit better to begin with (e.g. Fig 1). We do think that the result shown is correct because although PrP^Sc^ levels are low, they are maintained throughout the three successive rounds of propagation. After receiving this critique, we did attempt to repeat the experiment using PrP^C^ reconstituted with RNase-treated Prnp^0/0^ brain homogenate (rather than crude brain homogenate) to get better amplification, but were forced to halt the experiment before it could be completed because all of the research labs at Dartmouth were ordered to shut down until May 1 (in response to the COVID-19 crisis). We did previously publish a more convincing blot with similar results using a reconstituted reaction with mouse PrP^C^ and RNase-treated Prnp^0/0^ brain homogenate seeded with RML (Biochemistry. 2010 May 11;49(18):3928-34).*

*To provide perspective about this technical difficulty to the reader, we have added the following explanation to the Fig 2 caption:*

*“Note that the PMCA products in this experiment appear generally weaker compared to other experiments (e.g. Fig 1). This is most likely because the crude brain homogenate substrates (including mock-treated samples) were subjected to pre-incubation at 37oC prior to addition of seed, allowing endogenous enzymes to partially degrade and/or misfold PrP^C^ substrate. Nonetheless, all of the mock-control samples appear to successfully maintain the lower levels of PrP^Sc^ over three successive PMCA rounds.”*

2. The authors’ main thesis is that prion conformation, rather than sequence, is what controls cofactor and glycoform selection. However, a striking feature in the data overall is that all of the murine prion strains that were tested behave one way in terms of cofactor preference (preferring PE) while the hamster prion strains behave another (needing RNA). This would suggest that the PrP sequence of the prion (i.e. mouse vs hamster), in addition to different conformers thereof, are highly influential. If, as the authors argue, sequence doesn’t matter (much), why can none of the mouse strains (with their different conformations) use RNA as a cofactor for propagation, while both of the hamster strains can?

*This is an interesting point, and we have added the following possible explanation:*

*Although PrPSc strain conformation appears to play the dominant role in cofactor and glycoform selection for the versatile bank vole PrPC substrate, it seems likely that the primary sequence of PrPC could play a more important role in limiting cofactor and glycoform usage other species. This could explain why all 5 of mouse strains that we studied appear to be resistant to RNase treatment, whereas both of the hamster strains tested were RNase-sensitive. Interestingly, bank vole PrPC is natural chimera of the mouse and hamster PrPC sequences, and it is possible that some of the residues that differ between the mouse and hamster sequences are responsible for facilitating specific cofactor and glycoform preferences.*

3. L210: check spelling

*Thank you for pointing out this accidental error, which has been corrected.*

4. L293: Suggest Lawson & Priola, EMBO J (2001) as a primary reference here.

*Thank you for pointing out this appropriate and important reference, which has been added.*

**Reviewer #3:**

1. The authors state that distinct prion strains (RML and 139H) faithfully propagate in the presence of cofactors that are specific for each strain. However, prion strains are operationally defined by differences in heritable phenotypes under controlled experimental setups, like tissue tropism, incubation time and lesions profile. The authors should inoculate the PMCA products amplified in the presence of the specific cofactors into bank voles, to show that they retain the same lesion profiles of the original seeds.

*We only intended to propose a theoretical model that could potentially neurotropism, and did not mean to imply that any of the cofactor molecules currently identified are the preferred cofactors for any specific prion strain. It is not likely that either PE and RNA (the only cofactor molecules that we have identified to date) would be the strain-specifying cofactor molecules for RML or 139H, respectively. Therefore, we would not predict that the PMCA products formed with these cofactors would retain the same lesion profiles as the original seeds. Indeed, we previously performed a similar experiment with RML, Me7, and 301C in mice, and demonstrated that sPMCA propagation with pure PE causes strain restriction and the formation of a novel strain (Deleault, et al. PNAS 2012), whereas sPMCA propagation in crude brain homogenate maintains original strain properties (Castilla et al. Embo J. 2008).*

*We apologize for not making it clearer that the cofactor selection model is still speculative. To help clarify this point, we have added (1) the term “in vitro” to the title and (2) the following passage to the discussion section: “To test the cofactor selection model, it will be necessary to first identify cofactor molecule(s) that are able to maintain the strain properties of specific prion strains. In prior work, we showed that PE alone cannot maintain the strain properties of RML, Me7, or 301C[16].”*

2. The authors propose that the phenomenon of neurotropism could be explained by the preference of PrPSc seed for specific cofactors that are enriched only in some brain regions. I think that, although it might be a reasonable hypothesis, the data are not strong enough to support this claim. If feasible, the authors could replicate some of their experiments (i.e. PMCA amplification of seeds with and without RNAse treatment) using only some specific regions of the bank vole brains, instead of the whole brain homogenate, for example the brain region where these specific strains are known to preferentially propagate.

*Again, we proposed the cofactor selection model only to provide a theoretical framework for our findings, provide a possible explanation for neurotropism, and help guide future research directions. As explained above, it is not likely RNA is the strain-specific endogenous cofactor molecule for any of the prion strains that we studied. To test the model, we will have to first identify the cofactor molecule(s) needed to maintain strain properties for a specific prion strain.*

3. The authors mention a very interesting point (Discussion, Lines 234-243) about how cofactors determine the preference in conversion of prion seeds, and propose that molecules serving as cofactors for one prion strain may actually inhibit conversion of another strain. It would further strengthen the mechanism of this study if validation on this hypothesis could be performed via relative experiments, while it’s not contained in the required points.

*We have added a new Supplementary Figure S5 showing that RNA inhibits propagation and/or forces conformational adaptation of mouse recombinant PrP^Sc^ made with phospholipid cofactor. This result complements the referenced work from Ed Hoover’s group showing that phospholipid molecules inhibit CWD amplification by RT-QuIC.*

4. In Fig.3, the 1st passage of RML seed for Mo PrPC substrate shows a weak propagation of PrPSc in the “+ RNA group”, which could also be found in “-cofactor” groups for 1st passages of 139H seed for Ha PrPC, RML seed for BV PrPC, and 139H seed for BV PrPC. Please discuss the possible reasons.

*Unexpected Day 1 amplification is most often due to the presence of endogenous cofactor molecules in the prion-infected brain homogenates used as seeds for the first round of sPMCA. We have added this explanation to the caption for Fig 3.*
